# Gene expression-based comparison of the human secretory neuroepithelia of the brain choroid plexus and the ocular ciliary body: *potential implications for glaucoma*

**DOI:** 10.1186/2045-8118-11-2

**Published:** 2014-01-29

**Authors:** Sarah F Janssen, Theo GMF Gorgels, Jacoline B ten Brink, Nomdo M Jansonius, Arthur AB Bergen

**Affiliations:** 1 Department of Clinical and Molecular Ophthalmogenetics, the Netherlands Institute for Neuroscience (NIN), Royal Netherlands Academy of Arts and Sciences (KNAW), Meibergdreef 47, Amsterdam 1105 BA, The Netherlands; 2 Department of Ophthalmology, University of Groningen, University Medical Center Groningen, Groningen, The Netherlands; 3 Department of Clinical Genetics, AMC, Amsterdam, The Netherlands

## Abstract

**Background:**

The neuroepithelia of the choroid plexus (CP) in the brain and the ciliary body (CB) of the eye have common embryological origins and share similar micro-structure and functions. The CP epithelium (CPE) and the non-pigmented epithelium (NPE) of the CB produce the cerebrospinal fluid (CSF) and the aqueous humor (AH) respectively. Production and outflow of the CSF determine the intracranial pressure (ICP); production and outflow of the AH determine the intraocular pressure (IOP). Together, the IOP and ICP determine the translaminar pressure on the optic disc which may be involved in the pathophysiology of primary open angle glaucoma (POAG). The aim of this study was to compare the molecular machinery of the secretory neuroepithelia of the CP and CB (CPE versus NPE) and to determine their potential role in POAG.

**Methods:**

We compared the transcriptomes and functional annotations of healthy human CPE and NPE. Microarray and bioinformatic studies were performed using an Agilent platform and the Ingenuity Knowledge Database (IPA).

**Results:**

Based on gene expression profiles, we found many similar functions for the CPE and NPE including molecular transport, neurological disease processes, and immunological functions. With commonly-used selection criteria (fold-change > 2.5, *p*-value < 0.05), 14% of the genes were expressed significantly differently between CPE and NPE. When we used stricter selection criteria (fold-change > 5, *p*-value < 0.001), still 4.5% of the genes were expressed differently, which yielded specific functions for the CPE (ciliary movement and angiogenesis/hematopoiesis) and for the NPE (neurodevelopmental properties). Apart from a few exceptions (e.g. *SLC12A2, SLC4A4, SLC4A10, KCNA5,* and *SCN4B*), all ion transport protein coding genes involved in CSF and AH production had similar expression profiles in CPE and NPE. Three POAG disease genes were expressed significantly higher in the CPE than the NPE, namely *CDH1, CDKN2B* and *SIX1*.

**Conclusions:**

The transcriptomes of the CPE and NPE were less similar than we previously anticipated. High expression of CSF/AH production genes and candidate POAG disease genes in the CPE and NPE suggest that both might be involved in POAG.

## Background

Neuroepithelial cells, which themselves are derived from embryonic stem cells, can be viewed as neural progenitor cells [1,2]. They appear during embryonic development and form the neuroectoderm of the neural tube [3]. They give rise to multiple cell types, such as neurons, astrocytes and oligodendrocytes. In addition, in specialized areas of the central nervous system (CNS), these cells retain their epithelial nature and differentiate into fluid-secreting cells: the neuroepithelia of the ocular ciliary body and the choroid plexus epithelium of the brain [4-9]. These tight single-cell layers function together with the outer blood-retina barrier and the blood–brain barrier to maintain CNS homeostasis and are implicated in diseases like age-related macular degeneration, primary open-angle glaucoma (POAG) and Alzheimer’s disease.

In the literature, transcriptome analysis and functional predictions of human neuroepithelia, including the human retinal pigment epithelium [10] and ciliary body [11] in the eye and the choroid plexus epithelium (CPE) of the brain [12] have been described, but most studies are difficult to compare since they used different sampling methods, methodologies and bioinformatic techniques. Previously we determined the RNA expression profiles and predicted functional properties of five adult human neuroepithelia using a single microarray and bioinformatics platform: the central and peripheral retinal pigment epithelium [13-15], the pigmented and non-pigmented neural epithelia of the ocular ciliary body (CB) [16], and the choroid plexus neural epithelium (CPE) of the brain [17].

In the current study, we set out to compare the gene expression profiles and functional annotations of two, apparently closely related neural epithelia: the CPE, which produces the cerebrospinal fluid (CSF) of the brain and the CB epithelium (CBE), which produces aqueous humor (AH) of the eye.

The CPE and CBE resemble each other in many ways, as reviewed by Straziella and Ghersi-Egea [18] for the CPE and by Coca-Prados and Escribano [19] for the CBE. In summary, both epithelia are lobed neuroectodermal cell layers sealed by tight junctions, thereby creating diffusion barriers for most hydrophilic molecules between the blood and CNS. Both CPE and CBE produce colorless fluids, the CSF and AH, respectively. The CPE and CBE regulate production, composition and reabsorption of these fluids and are involved in intra-cranial and intra-ocular pressure dynamics, respectively [20,21]. Both the CPE and CBE form villi that overly a loose extracellular matrix and fenestrated choroidal capillaries.

There are also major differences between the CPE and CBE. Obviously, they are located in different parts of the CNS: the CPE is located in the lateral, third and fourth ventricles of the brain, whereas the CBE resides in the posterior chamber of the eye. A major difference is that the CBE consists of two adjacent neuroectodermal layers: the non-pigmented (NPE) and the pigmented epithelium (PE), whereas the CPE consists of a single epithelial layer. Using large scale gene expression analysis and functional annotation, we recently showed that the NPE and PE layers are highly comparable: only 1% of the transcriptome was different between the NPE and PE [16].

The CPE and CBE are involved in the pressure dynamics in the brain and eye, respectively. The pressures of the ocular and cranial fluids uniquely meet at one location: the optic disc, situated at the point where the optic nerve leaves the eye. The optic disc contains the lamina cribrosa that supports the retinal nerve fibers where they leave the eye. The difference between the posteriorly directed IOP and anteriorly directed ICP on the optic disk is the translaminar pressure gradient. This translaminar pressure ultimately determines the forces applied to the lamina cribrosa and these forces appear to play a key role in a common age-related eye disease POAG. POAG is a progressive optic neuropathy with abnormal cupping of the optic nerve head (excavation) and loss of retinal ganglion cells. The pathogenesis of POAG is largely unknown, but an increased IOP is a major risk factor. Recent findings suggest that it is actually the translaminar pressure, rather than the IOP, that forms the risk factor for POAG and its subtype normal tension glaucoma (NTG) [22-29].

Since the CPE and CBE have a similar origin, structure and function, and together are involved in translaminar pressure dynamics, we were interested in the gene expression-based functional comparison of the human CPE and CBE. Previously, we found that the gene expression of the two ciliary epithelia, PE and NPE, resembled each other closely [16]. For the present study we chose to compare the CPE with the NPE (and not the PE), since the NPE is non-pigmented and contains tight junctions, just like the CPE. The aims of this study were (1) to compare the gene expression profiles and functional annotation of the CPE and NPE in order to determine common properties and potential specific functions and (2) to determine CPE/NPE highly and specifically expressed genes coding for ion transport proteins involved in CSF/AH production and (3) for genes previously associated with POAG.

## Methods

### Ethics statement

This study was performed after institutional approval of the Netherlands Institute for Neuroscience, Amsterdam, Netherlands. The human CPE material was obtained from the Netherlands Brain Bank (Amsterdam, Netherlands). The human donor eyes were provided by the Corneabank Beverwijk, Netherlands. In accordance with the international declaration of Helsinki, the NBB and the Corneabank obtained permission from the donors for, respectively, brain and eye autopsy and the use of clinical information for research purposes. All human data were analyzed anonymously.

### Gene expression data of the human choroid plexus and ciliary body epithelia

We used the gene expression data from our previously-published microarray studies of healthy human CPE (seven samples) (GSE49974; [17]) and NPE (seven samples) (GSE37957; [16]). The age of the donors varied between 39 and 73 years. Donors had neither history of any brain or eye disease nor any malignancies. The seven human CPE donors were all male, five human CBE donors were male and two female. Since several external or genetic factors may have influence on our human gene expression data, we could detect only consistent and significant similarities or differences between the selected samples. All RNA samples from each tissue were analyzed separately by microarray. The microarrays were performed against the same common reference sample, being human retinal pigment epithelium (RPE)/choroid. In this way, the CPE and NPE gene-expression data could be compared with each other. Human choroid plexus tissue was removed from the lateral ventricles of fresh post mortem brains. Whereas the gross anatomy of the CPE became somewhat distorted, the neuroepithelium could be identified easily on cryosections for laser dissection. Donor eyes were snap-frozen in their entirety, which maintained the overall structure of the ciliary body. Detailed description of cell sampling, RNA processing, microarray hybridization, and confirmation methodology of the microarray data were described elsewhere previously [16].

### Data analysis

The microarray image files were analyzed and processed by Agilent Feature Extraction Software (Agilent Technologies, version 9.5.3.1) and mean intensities (log2) were given to the spots. These mean intensities were normalized between arrays of the CPE and NPE in R (version 2.14.0 for Windows, R Development Core Team, 2009; NormalizeBetweenArrays with the ‘aquantile’ method). Next, the genes were ranked by expression level and assigned percentile ranks [13]. The expression datasets were functionally annotated using the Ingenuity Knowledge Base (Ingenuity® Systems, version 11631407, http://www.ingenuity.com; assessed at August 27, 2013). The ingenuity core-analysis yields information about biological functions, functional molecular networks, and canonical pathways. Functional molecular networks represent the interactions of molecules leading to biological functions that are attributed to a certain dataset of genes. Canonical pathways are the simplest representation of an (established) molecular pathway. The Ingenuity database contains millions of molecular and functional data points from experimental studies, available from both literature and online experimental databases such as PUBMED, the GWAS Database, GEO, and OMIM.

For the first functional analysis, genes were selected with the highest expression values (expression above 90th percentile [P90]) in both the CPE and NPE. This means that these genes have an expression intensity that falls into the highest 10% intensity values of the CPE and NPE microarrays [13,16]. These genes are referred to as the “Highly expressed CPE and NPE genes”. Next, the gene expression data of the CPE and NPE was compared with a t-test in R and Benjamini-Hochberg correction for multiple testing (version 2.14.0 for Windows, R Development Core Team, 2009). Our initial selection criteria for differentially expressed genes were a fold-change (FC) >2.5 and a *p*-value <0.05. To identify larger and more specific differences, a relatively strict criteria of FC > 5 and a *p*-value < 0.001 was used, and this resulted in a set of genes referred to as the “Significantly differentially expressed CPE and NPE genes”. These significantly differentially expressed CPE and NPE genes were functionally annotated in the Ingenuity Knowledge Base (Ingenuity® Systems, version 11631407, http://www.ingenuity.com; assessed at August 5, 2013). In addition, we looked for highly and significantly differentially expressed ion transport protein coding genes involved in CSF/AH production in the CPE and NPE. The ion-channels and ion transporters that are involved in CSF and AH production were, respectively, reviewed by Brown *et al*. [30] and Civan *et al*. [20]. Ingenuity was searched for corresponding genes that code for these channels and transporters. In this way, we created a list of genes involved in CSF/AH production. Next, this list was compared with the “Highly expressed CPE and NPE genes” and “Significantly differentially expressed CPE and NPE genes” to find common or different features in CPE and NPE regarding CSF/AH production. Finally, we searched for (candidate) POAG disease genes that were highly or significantly differentially expressed in the CPE and NPE. To this end we compared the “Highly expressed CPE and NPE genes” and “Significantly differentially expressed CPE and NPE genes” with a list of 65 (candidate) POAG disease genes that we recently reviewed [31].

## Results

### Comparison of the CPE and NPE transcriptome

There were several functions that are apparently biologically important in both epithelial layers. These were (1) neurological function and disease (migration of neuroglia, Alzheimer’s disease, Parkinson’s disease, tauopathy, Leigh syndrome), (2) immunological and infectious diseases (allergy and autoimmune disease, viral infection pathways), (3) molecular transport, (4) hematological disease (myelodysplastic syndrome, lymphosarcoma, anemia), and (5) basic cellular (dys) functions. However, it is possible that similar expression of house-keeping genes in both tissues underlie some of these shared functional annotations.

The statistical comparison between the entire gene expression datasets of 30,589 unique genes of the CPE and NPE showed that many genes were also significantly differentially expressed between the CPE and NPE. Surprisingly, with the frequently used standard ANOVA selection criteria (FC > 2.5 and *p*-value < 0.05; [15,16]), far more significantly differentially expressed genes (14%) were found than we had expected *a-priori*. To identify the most important and specific differences, we subsequently chose a more strict selection criteria (FC > 5 and *p*-value < 0.001). With these criteria, there were 760 (2.5%) genes significantly more highly expressed in the CPE than in the NPE. *Vice versa*, there were 624 (2.0%) genes significantly more highly expressed in the NPE out of a total of 30,589 unique genes. Tables 1 and 2 present the top-30 of “Significantly differentially expressed CPE and NPE genes” according to the strict criteria. The complete lists of the expressed genes in CPE and NPE can be found in Additional files 1 and 2, respectively.

**Table 1:** Top-30 genes significantly higher expressed in CPE compared to NPE

**Gene name**	**Systematic name**	**FC**	***p*-value**
*TTR*	NM_000371	4307.5	2.38E-12
*SLC24A4*	NM_153646	425.1	3.49E-14
*W60781*	W60781	390.8	4.65E-09
*ARMC3*	NM_173081	196.7	7.12E-13
*KL*	NM_153683	191.7	4.53E-10
*SPAG6*	NM_012443	171.5	2.74E-11
*SLCO1C1*	NM_017435	134.0	1.96E-11
*KLK11*	NM_144947	127.7	1.47E-14
*IL13RA2*	NM_000640	97.5	4.59E-12
*FAM81B*	NM_152548	90.5	2.08E-11
*EFCAB1*	NM_024593	88.1	4.38E-13
*THC2741529*	THC2741529	82.8	1.86E-09
*DNER*	NM_139072	82.6	2.08E-12
*KIAA1456*	NM_020844	77.6	2.09E-12
*C13orf26*	NM_152325	77.5	1.13E-12
*SLC4A10*	NM_022058	73.1	9.42E-10
*FLJ23049*	NM_024687	71.8	3.49E-12
*KCNA5*	NM_002234	70.4	1.97E-11
*NAT8L*	NM_178557	69.9	5.20E-10
*SPAG8*	NM_172312	62.9	1.47E-10
*BUB1B*	NM_001211	59.2	7.40E-08
*AIM2*	NM_004833	55.6	9.11E-13
*THC2657259*	THC2657259	55.0	6.28E-11
*GRM8*	NM_000845	54.9	5.38E-10
*HRK*	NM_003806	54.6	8.93E-11
*POLE*	AF128542	54.3	4.24E-09
*FABP4*	NM_001442	52.4	8.89E-10
*C2orf39*	NM_145038	50.6	2.09E-12
*CLIC6*	NM_053277	49.7	2.07E-11
*KIAA1199*	NM_018689	47.9	1.10E-12

**Table 2:** Top-30 genes expressed significantly higher in NPE compared to CPE

**Gene name**	**Systematic name**	**FC**	**p-value**
*AOC3*	NM_003734	375.2	4.24E-10
*FGFBP2*	NM_031950	272.4	9.92E-11
*THC2677285*	THC2677285	268.8	9.11E-13
*LOC92196*	NM_001017920	250.9	3.32E-11
*CPAMD8*	NM_015692	214.9	9.80E-11
*HSD17B2*	NM_002153	195.9	9.23E-12
*CD96*	NM_198196	162.3	9.23E-12
*AK124698*	AK124698	161.5	7.00E-10
*GRM1*	NM_000838	128.9	2.64E-13
*PCSK2*	NM_002594	125.6	2.49E-11
*CLRN1*	NM_174878	118.3	1.09E-12
*LIPC*	NM_000236	107.8	2.40E-12
*ATP1A2*	NM_000702	105.1	2.77E-08
*C8orf47*	NM_173549	89.4	2.38E-12
*TFPI2*	NM_006528	88.7	2.44E-09
*DSC1*	NM_004948	87.4	1.20E-10
*GPR64*	NM_001079858	86.5	8.89E-12
*SLC5A8*	NM_145913	85.6	5.97E-14
*LHX2*	NM_004789	77.8	2.64E-13
*RAX*	NM_013435	77.7	1.70E-11
*ABI3BP*	NM_015429	76.6	1.65E-09
*FAM83D*	NM_030919	75.9	4.79E-12
*BHLHB5*	NM_152414	75.4	5.31E-10
*SLITRK5*	NM_015567	66.0	5.56E-10
*THC2636507*	THC2636507	64.2	1.45E-08
*THC2676797*	THC2676797	62.6	7.23E-12
*DSCR8*	NM_203429	61.8	2.38E-12
*OXGR1*	NM_080818	60.7	4.41E-11
*LRRN1*	NM_020873	56.7	9.48E-12
*KIAA1727*	NM_033393	55.6	3.40E-09

### Functional annotation of the genes expressed significantly higher in CPE than in NPE

**Figure 1:**
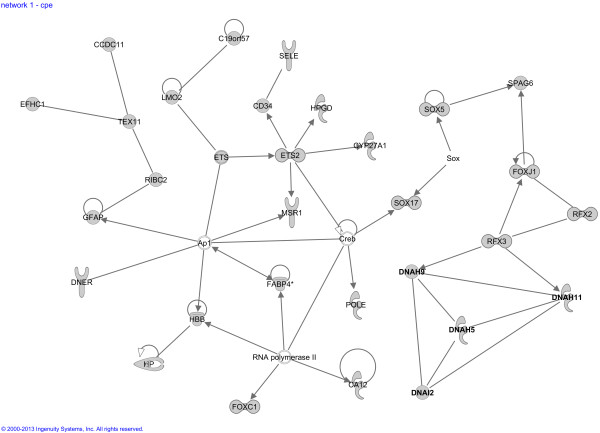
**Molecular network of genes expressed significantly more highly in the brain choroid plexus epithelium compared to the non-pigmented epithelium of the eye.** Example of a molecular network generated by Ingenuity software. Grey symbols represent genes expressed more highly in the CPE than in the NPE. Transparent entries are molecules inserted by the knowledge database. Gene names are abbreviated according to those used in GenBank. Solid lines between molecules indicate direct physical or functional relationships between molecules (such as regulating and interacting protein domains). The genes *DNAH5, DNAH9, DNAH11* and *DNAI2* indicated in bold, code for dynein proteins. Dyneins keep the microtubules of the cilia together and promote the movement of the cilia. Mutations in these genes can cause primary ciliary dyskinesia. The main functionalities given by Ingenuity for this entire molecular network are “Developmental and hereditary disorder and respiratory disease”. Reproduced with permission.

Ingenuity attributed four major statistically significant biological functions to the CPE: (1) ciliary and flagella motion and primary ciliary dyskinesia, (2) development of blood vessels and angiogenesis and related diseases (vascular disease, occlusion of artery, arteriosclerosis), (3) molecular transport, and (4) several types of cancer (adenocarcinoma, epithelial neoplasia, cancer of lung, uterine endometrium, and ovary). Using the same dataset, Ingenuity subsequently built 25 functional molecular CPE networks*.* Figure 1 shows as an example, a diagram of network 1 that represents the gene and protein interactions leading to ciliary movement and dyskinesia. This network is functionally annotated with “Developmental and hereditary disorders and respiratory disease”. The other 24 functional molecular network diagrams can be found in Additional file 3. After correction for multiple testing, there were no statistically significant canonical pathways specifically assigned by Ingenuity to the CPE compared with the NPE.

### Functional annotation of the genes expressed significantly higher in NPE than in CPE

Ingenuity assigned four major statistically significant biological functions to the NPE compared to the CPE. The first and most significant biological function was (1) nervous system development, and more specifically neuronal development of sensory organ, head, body axis, eye, forebrain, brain, and telencephalon. This functionality also comprised neurological and ophthalmic diseases (seizure, epilepsy, schizophrenia, bipolar disorder, and congenital anomalies of the eye). Examples of genes underlying this NPE functionality are *ALDH1A1, ATOH7, CYP1B1, MSX2, OTX1, PAX6, PVRL3, RP1, SEMA5A, SIX6,* and *SOX4* (eye development) and *BMP2, FGFR1, GAP43, GLI3, GSX2, HES1, PAX6, RELN, SLIT1, SOX3, SOX4,* and *ZIC3* (brain development). For the total list, see Additional file 4. Another statistically significant function was formation of cellular protrusions and plasma membrane projections. This function was based on 47 genes expressed significantly higher in NPE, for example genes involved in actin function (*ANK3, ARGHGAP24, ENC1, MARCKS, PALLD*), genes involved in filopodium formation and cytoskeleton organization (*MCF2, RHOU*) and axonal growth and guidance (*CNTN4, GAP43, LRRC4C, SEMA3A, TIAM1, TIAM2*). For the total list, see Additional file 5. Finally, we identified the functions of molecular transport, and several types of cancer (adenocarcinoma, epithelial neoplasia, cancer of lung).

**Figure 2:**
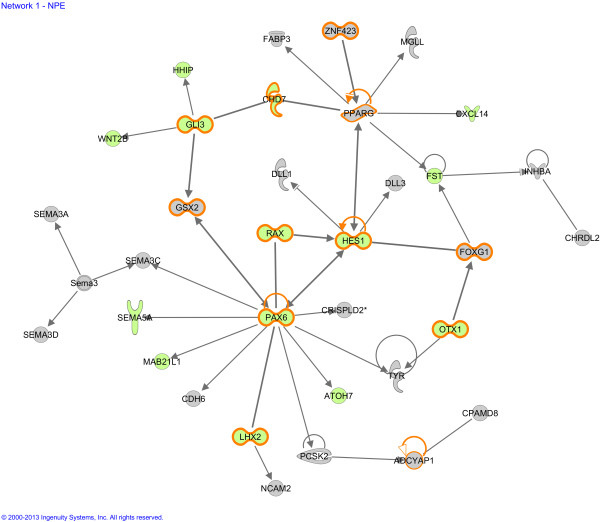
**Molecular network of genes expressed significantly more highly in non-pigmented epithelium of the eye compared to the brain choroid plexus epithelium.** Example of a molecular network generated by Ingenuity software. Gene names are abbreviated according to those used in GenBank. Solid lines between molecules indicate direct physical or functional relationships between molecules (such as regulating and interacting protein domains). This network contains many genes involved in brain and nervous system development (orange bordered symbols) and eye development (green filled symbols)*.* The main functionalities given by Ingenuity for this entire molecular network are “Embryonic organ and organismal development”. Reproduced with permission.

Subsequently, from the same dataset, Ingenuity built 25 functional molecular networks significantly present in the NPE, but not in the CPE. Figure 2 presents, as an example, a diagram of network 1 that shows specific neurodevelopmental functions of the NPE, annotated with top functions “Embryonic, organ and organismal development”. The other 24 functional molecular network diagrams can be found in Additional file 6. After correction for multiple testing, Ingenuity did not assign statistically significant canonical pathways specific for the NPE compared to the CPE.

### CPE and NPE expressed genes involved in CSF and AH production

We constructed a list of 157 coding genes corresponding to all the CPE and CBE ion channels and ion transporters proteins as described by Brown *et al.*[30] and Civan *et al.*[20] (see Additional file 7). Table 3 presents a list of ion channels and ion-transporters of which at least some of the coding genes were highly and/or significantly differentially expressed in the CPE and NPE, in order to find common or different features in CPE and NPE regarding CSF/AH production. Based on our gene expression data, all these ion channels and ion transporters were present in both epithelia. Eight coding genes were both highly expressed in CPE and NPE (*SLC12A7, ATP1A1, ATP1A2, ATP1B1, ATP1B2, ATP1B3, KCNJ13,* and *AQP1*), 13 were expressed significantly higher in the CPE compared to the NPE (*ATP1B1, SLC12A2, SLC4A2, SLC4A10, KCNA5, KCNE1, KCNF1, KCNH2, KCNK1, KCNN2, KCNN3, SCN2A,* and *AQP4*), and 10 higher in the NPE compared to the CPE (*ATP1A1, ATP1B3, SLC4A4, KCNA4, KCNB2, KCNG1, KCNS1, SCN2B, SCN3B,* and *SCN4B*).

**Table 3:** Genes coding for ion channels and transporters involved in CSF and AH production in the CPE and NPE

**Ion channels and transporters**	**Present in**	**Coding genes**	**High gene expr.**	**Sign. diff. gene expr.**
Na^+^/K^+^ ATPase	CPE and CBE	*ATP1A1*	Both	NPE
		*ATP1A2*	Both	
		*ATP1A3*	CPE	
		*ATP1A4*		
		*ATP1B1*	Both	CPE
		*ATP1B2*	Both	
		*ATP1B3*	Both	NPE
Na^+^/2Cl^-^/K^+^ co-transport	CPE and CBE	*SLC12A1*		
		*SLC12A2*	CPE	CPE
Na^+^/Cl^-^ symport	CBE	*SLC12A3*		
K^+^/Cl^-^ symport	CPE	*SLC12A4*		
		*SLC12A5*		
		*SLC12A6*		
		*SLC12A7*	Both	
		*SLC12A9*		
Cl^-^/HCO3^–^ exchanger	CPE and CBE	*SLC4A1*		
		*SLC4A2*		CPE
		*SLC4A3*		
Na^+^/HCO3^–^ symport	CPE	*SLC4A4*	NPE	NPE
		*SLC4A5*		
		*SLC4A7*		
		*SLC4A8*		
		*SLC4A9*		
		*SLC4A10*	CPE	CPE
Na^+^/H^+^ exchanger	CPE and CBE	*SLC9A1*		
		*SLC9A2*		
		*SLC9A3*		
		*SLC9A4*		
		*SLC9A5*		
		*SLC9A6*		
K^+^ channel	CPE and CBE	*KCNA4*		NPE
		*KCNA5*	CPE	CPE
		*KCNAB1*	NPE	
		*KCNB2*		NPE
		*KCNE1*		CPE
		*KCNF1*		CPE
		*KCNG1*		NPE
		*KCNH2*		CPE
		*KCNJ13*	Both	
		*KCNK1*	CPE	CPE
		*KCNN2*		CPE
		*KCNN3*		CPE
		*KCNS1*		NPE
Cl^-^ channel	CPE and CBE	*CLCN1*		
		*CLCN2*		
		*CLCN3*		
		*CLCN4*		
		*CLCN5*		
		*CLCN6*		
		*CLCN7*		
Na^+^ channel	CPE and CBE	*SCN1A*		
		*SCN1B*		
		*SCN2A*		CPE
		*SCN2B*		NPE
		*SCN3A*		
		*SCN3B*		NPE
		*SCN4A*		
		*SCN4B*	NPE	NPE
		*SCN5A*		
		*SCN7A*		
		*SCN8A*		
		*SCN9A*		
		*SCN10A*		
		*SCN11A*		
Water channel	CPE and CBE	*AQP1*	Both	
		*AQP2*		
		*AQP3*		
		*AQP4*		CPE
		*AQP5*		
		*AQP7*		
		*AQP8*		
		*AQP9*		
		*AQP10*		
		*AQP11*		

This table lists the ion channels and ion transporters involved in cerebrospinal fluid (CSF)/aqueous humor (AH) production, their presence in choroid plexus epithelium (CPE) and/or non-pigmented epithelium (NPE) of the ciliary body epithelium according to literature [20,30] and the gene expression profiles in the CPE and NPE according to our microarray gene expression studies. Column 1: Ion channels and transporters involved in CSF/AH production. For the K^+^ channel, we only presented the highly and/or statistical significant different expressed genes of the CPE and NPE; Column 2 - Present in: The presence of the different ion channels and ion transporters are reviewed in [30] for the CPE and [20] for the NPE; Column 3 - Coding genes: the genes coding for the ion channels and ion transporters listed in column 1; Column 4 - High gene expr.: Here we indicate the genes that are highly expressed in the CPE and/or NPE according to our gene expression microarray data of the CPE and NPE. Highly expressed gene are genes with mean expression values >90th percentile. This means that these genes have an expression intensity that falls into the highest 10% intensity values of the microarray. Both = high expression in both CPE and NPE; Column 5 - Sign. diff. gene expr.: Here we indicate the genes that were statistically significant higher expressed in the CPE or NPE compared with the NPE and CPE, respectively, according to our gene expression microarray data of the CPE and NPE. The selection criteria were fold change > 5 and *p*-value < 0.001.

### CPE and NPE expressed genes previously associated with POAG

We compared a recently constructed list of 65 candidate POAG genes (reviewed in [31]) with the genes that were highly and/or significantly differently expressed in the CPE and NPE. Table 4 presents the results. We found 10 candidate POAG genes that were highly expressed in both the CPE and NPE (*AKAP13, C1QBP, CHSY1, COL8A2, CYP1B1, FBN1, IBTK, MFN2, TMCO1,* and *TMEM248),* three genes that were expressed significantly higher in the CPE (*CDH1, CDKN2B,* and *SIX1*), and six genes that were expressed significantly higher in the NPE (*ATOH7, CYP1B1, FBN1, MYOC, PAX6,* and *SIX6*).

**Table 4:** Gene expression profiles of candidate POAG disease genes in CPE and NPE

**Candidate POAG disease genes**	**High gene expr.**	**Sign. diff. gene expr.**
*APOE*	CPE	
*AKAP13*	Both	
*ATOH7*		NPE
*C1QBP*	Both	
*CDH1*	CPE	CPE
*CDKN2B*		CPE
*CHSY1*	Both	
*COL8A2*	Both	
*CYP1B1*	Both	NPE
*FBN1*	Both	NPE
*GSTM1*	CPE	
*IBTK*	Both	
*MFN2*	Both	
*MYOC*	NPE	NPE
*OPTN*	CPE	
*PAX6*	NPE	NPE
*RFTN1*	NPE	
*SIX1*		CPE
*SIX6*	NPE	NPE
*TMCO1*	Both	
*TMEM248*	Both	

High gene expr: Genes that are highly expressed in the CPE and/or NPE according to our gene expression microarray data of the CPE and NPE. Highly expressed gene are genes with mean expression values >90th percentile. Both = high expression in both CPE and NPE. Sign. diff. gene expr.: Genes that are statistically significant higher expressed in the CPE or NPE compared with the NPE and CPE, respectively, according to our gene expression microarray data. The selection criteria were fold change > 5 and *p*-value < 0.001.

## Discussion

The gene expression data of the brain CPE and the NPE of the CBE from the eye were compared. The CPE and NPE are both secretory neuroepithelia, which share a common embryological origin and important structural and functional features (fluid production, brain and eye homeostasis and pressure dynamics) but also exhibit substantial anatomical differences (location, single (CPE) or double (CBE) layered). Together, the CPE and CBE are ultimately involved in building the translaminar pressure of CSF and AH on the optic disc. A disturbed translaminar pressure is a risk factor for POAG and NTG.

### Comparison of the CPE and NPE transcriptome

It had been anticipated that the transcriptomes and predicted functionalities of the CPE and NPE in the current study would be highly similar. After all, using (initially) the same criteria, methodology and platform, the neuroepithelial transcriptomes of the ocular NPE and PE differed only by 1% [16] and the transcriptomes of the macular and peripheral RPE only by 1-5% [15]. Several overlapping functionalities for the CPE and NPE most highly expressed genes were found, including neurological function and diseases, immunological properties, and molecular transport. However, unexpectedly, there were also large differences between the two neuroepithelia. In the initial analysis, in which we applied the same selection criteria (FC > 2.5 and *p*-value < 0.05) as used in our previous neuroepithelial microarray studies [13,15,16], there was a large, 14% difference (data not shown); in our final analysis (FC > 5 and *p*-value < 0.001) this was still 4.5%. Hence, although the transcriptomes of the two neuroepithelia showed substantial overlap, there were large differences as well.

Previously published data showed many molecular similarities between CPE and CBE, but also some differences: for example the ion channels and ion transporters involved in CSF and AH production. These were widely studied and extensively reviewed by Brown *et al.*[30] for the CPE and by Civan *et al*. [20] for the CBE. These reviews reported that most of the ion channels and ion-transporters involved in the CSF/AH production are found in both the CPE and CBE, for example Na^+^/K^+^-ATPase, Na^+^/K^+^/2Cl^-^ -symporters, Cl^-^/HCO3^–^ and Na^+^/H^+^ exchangers, and aquaporins. However, there were also some ion transport proteins in the CPE that have not been identified in the CBE, for example Na^+^/HCO3^–^ and K^+^/Cl^-^ symporters. On the other hand, Na^+^/Cl^-^ exchangers were found in the CBE, but not (yet) in the CPE [20,30].

### Predicted CPE functionalities not present in the NPE

#### Function of cilia

The most important predicted function found in the CPE, but not in the NPE, was genes for ciliary motion and primary ciliary dyskinesia. Cilia, which consist of bundles of microtubules held together by dyneins, are essential for CSF flow in the brain ventricles. Hydrocephalus is a common sign of primary ciliary dyskinesia, a disease caused by mutations in dynein proteins [32,33]. A detailed inspection of the expression datasets showed that six dynein coding genes (*DNAH5, DNAH7, DNAH9, DNAH11, DNAI1,* and *DNAI2)* were expressed significantly more highly in the CPE compared to the NPE. *Vice versa*, there were no dynein coding genes expressed more highly in the NPE compared to the CPE. Hence, our data are in line with the idea that ciliary function is biologically more important in CPE than in NPE.

#### Angiogenesis

Another important CPE function that was not present in the NPE was “angiogenesis and development of blood vessels”. Ingenuity yielded this function because of 57 relevant genes expressed significantly higher in the CPE than in the NPE, most importantly *ACE2, ADRA2B, AHR, FN1, FOXC1, GATA6, HGF, HHEX, KLF2, PGF, PTHLH, SERPINF1,* and *TEK* (for the complete list, see Additional file 8). The appearance of this function could be explained either by a technical artifact or by a genuine biological reason. The CPE samples are much more closely entangled with blood vessels than the NPE. Thus, potential contamination of CPE tissue with blood vessel cells during laser dissection microscopy may underlie this finding. We previously showed that cellular contamination of adjacent tissue is a possible limitation of (our) cellular microarray approach [13]. Alternatively, the CPE could actually have a specific function that is not present in the NPE. This function could be extramedullary hematopoiesis (EH), which previously has been assigned to the human and mouse CPE [31]. Indeed, the functional molecular network 4 (“Hematological system development and function and hematopoiesis”) built by Ingenuity of significantly higher expressed CPE genes, also points to this specific property (Additional file 3). EH is a compensatory mechanism of hematopoiesis in sites other than the bone marrow, and some case reports point to a role of EH in the CPE [34-36]. We could not find published reports of possible presence of EH in the NPE.

### Predicted NPE functions not present in the CPE

#### Neuronal development

The first and most significant predicted function for the NPE not present in the CPE was “development of neuronal tissues and organs, including eye, retina, brain and forebrain”. The potential neural embryological properties of the CBE have been a topic of fierce recent discussion. The *pars plana* and the ciliary marginal zone of the CBE may, or may not, contain retinal progenitor cells, and there may even be differences between animal species. Some authors reported the presence of specific retinal stem cell or progenitor cell markers, such as NES, MITF, PAX6, SIX3, Rx, FGF2, and CHX10 [37-42]. Isolated CBE neural progenitor cells were shown to proliferate and differentiate *in vitro* into neural spheres and possible photoreceptor-like cells [37,43-50], although this was not found in all studies [51,52]. Interestingly, our gene expression data are primarily of the *pars plicata* of the human NPE, not from the *ora serrata*, but, even here, some embryological properties seem to be present in the adult neuroepithelium.

#### Cellular protrusions

The second predicted function of the NPE was “formation of cellular protrusions and plasma membrane projections”. The reason why this function is apparently more prominent in the NPE is currently not clear. After all, both the NPE and CPE contain actin and both cell types have filopodia. Possibly, the attachment of lens zonule fibers to the NPE may play a role in this finding. Future research is warranted.

### Ion channels and ion transporters involved in CSF and AH production

The genes coding for the ion channels and ion-transporters involved in CSF and AH production had highly similar expression patterns in both tissues. Indeed, in the literature, some of these proteins were already identified in the CPE and CBE, such as ATP1A1, ATP1B1 and ATP1B2 [20,30], AQP1 [53,54] and SLC12A7 [55]. However, there are some significant expression differences in specific genes, which point to (subtle) differences in CSF and AH production: namely 13 specifically-expressed genes in the CPE and 10 in the NPE. To compare, a study of the molecular background of AH production in the CBE, the NPE and PE only showed four specifically expressed genes in the PE and none in the NPE [56]. At the protein level, previous studies showed expression of SLC12A2 [57], SLC4A2 [58], and SLC4A10 [59] in the CPE and expression of SLC4A4 [60] and sodium channels [61] in CBE. However, these finding were not proof that these proteins definitely differ between CPE and CBE, and comparative studies on protein level in both CPE and CPE are necessary to determine this. The differences between CPE and NPE could be a starting point for further biochemical, physiological and pharmacological research. Perhaps, these differences will open the door for specific drug delivery to the brain and the eye after systemic treatment as for example in a systemic drug that simultaneously increases ICP and lowers the IOP for POAG/NTG treatment.

### Potential CPE and NPE involvement in POAG

Both the CPE and CBE may, ultimately, play a role in the development of POAG (see Background section), and especially in the development of NTG [31]. Ten (*AKAP13, C1QBP, CHSY1, COL8A2, CYP1B1, FBN1, IBTK, MFN2, TMCO1* and *TMEM248)* of the 65 candidate POAG disease genes were highly expressed in both CPE and NPE, which might indicate overlapping pathobiological mechanisms of POAG in the two epithelia. On the other hand, we also identified three candidate POAG disease genes, *CDH1, CDKN2B* and *SIX1,* which were expressed more highly in the CPE than in the NPE. CDKN2B regulates cell cycle and growth, SIX1 is involved in developmental processes and CDH1 is a cell-cell adhesion glycoprotein. Interestingly, mutations in *CDKN2B* were previously implicated in NTG [62-65]. In addition, mutations in both *CDKN2B* and *SIX1* were previously associated with the vertical-cup-disc-ratio, an endophenotype of POAG [66,67], and not, for example, with IOP. *Vice versa*, we found six (candidate) POAG-disease genes significantly higher expressed in the NPE than in the CPE *(ATOH7, CYP1B1, FBN1, MYOC, PAX6,* and *SIX6)*. The potential involvement of these genes in POAG via the CPE and CBE is currently not clear.

As mentioned in the Background section, it seems that not only an increase in IOP but also a decrease in ICP could be a risk factor for POAG [22-29,68-70]. Thus, manipulation of either IOP or ICP, together or apart, would be a possible form of POAG treatment. For example, alpha-adrenergic receptors are present in the both NPE and CPE albeit with differences in affinity and effect upon stimulation [71-73]. Our expression data show that *ADRA2B* (an alpha-2-adrenergic receptor) is expressed more highly in the CPE compared to NPE and *ADRA1B* (an alpha-1-adrenergic receptor) is expressed more highly in the NPE compared to the CPE. Of specific interest are the alpha-2 adrenoceptors in the brain and eye. Chiou *et al.*[71] found that intraocular administration of an alpha-2 antagonist (yohimbine) resulted in a decreased IOP in cats, whereas McCormick *et al.*[73] showed that an intravenous administered alpha-2 antagonist (tolazine) resulted in an increased ICP. These findings might suggest that an intravenous alpha-2 antagonist is an ideal treatment option for POAG and NTG: it lowers the IOP and increases the ICP, thereby changing the translaminar pressure on both sides. Chiou *et al.*[71] also found that intraocular treatment with an alpha-2 agonist (clonidine) resulted in a decreased IOP (but less effective than alpha-2 antagonist). In the brain, intravenous alpha-2 agonist (xylazine) resulted also in a decreased ICP, making this agent less interesting for the dual, systemic treatment of POAG/NTG. However, more research is warranted, to prove these findings and hypothesis, to specify which alpha-2 antagonist does both decrease IOP and increase ICP, and if this approach is a good treatment option.

## Conclusions

The gene expression patterns and predicted functions of the CPE and NPE overlap (molecular transport, immunological functions), but also show substantial differences. The ion channels and ion-transporters involved in CSF and AH production share the same expression profiles for most of their coding genes in CPE and NPE, with a few exceptions. The CPE showed specific functions in ciliary movement and angiogenesis/hematopoiesis compared to the NPE. *Vice versa*, the NPE analysis yielded specific neurodevelopmental properties not present in the CPE. In POAG and NTG, the CPE and NPE could act together in creating a disturbed, net posteriorly directed translaminar pressure over the optic disc, where brain and eye meet.

## Abbreviations

AH: Aqueous humor; EH: Extramedullary hematopoiesis; CBE: Ciliary body epithelium; CNS: Central nervous system; CPE: Choroid plexus epithelium; CSF: Cerebrospinal fluid; ICP: Intracranial pressure; IOP: Intraocular pressure; NPE: Non-pigmented epithelium; NTG: Normal tension glaucoma; PE: Pigmented epithelium; POAG: Primary open angle glaucoma; RPE: Retinal pigment epithelium.

## Competing interests

The authors declare that they have no competing interests.

## Authors’ contributions

SJ carried out the microarray studies and drafted the manuscript; TG participated in study design and helped to draft the manuscript; JB carried out the microarray studies; NJ participated in study design and helped to draft the manuscript; AB participated in study design and drafted the manuscript. All authors read and approved the final manuscript.

## Supplementary Material

Additional file 1 **Total list of genes expressed significantly higher in CPE compared to NPE.** Selection criteria were a fold change > 5 and *p*-value < 0.001. Click here for file

Additional file 2 **Total list of genes expressed significantly higher in NPE compared to CPE.** Selection criteria were a fold change > 5 and *p*-value < 0.001. Click here for file

Additional file 3 **Molecular networks 2–25 generated by the Ingenuity software from the genes expressed significantly higher in the CPE compared to NPE.** Grey symbols represent genes expressed significantly higher in the CPE. Transparent entries are molecules inserted by the knowledge database. Gene names are abbreviated according to those used in GenBank. Solid lines indicate direct physical or functional relationships between molecules (such as regulating and interacting protein domains). The main functionalities given by Ingenuity for this entire molecular network are shown in the diagrams. Reproduced with permission. Click here for file

Additional file 4 Genes in the NPE expressed significantly higher than in CPE involved in the biological function of development. Click here for file

Additional file 5 Genes in the NPE expressed significantly higher than in CPE involved in the biological function of cellular protrusions. Click here for file

Additional file 6 **Molecular networks generated by the Ingenuity software from the genes expressed significantly higher in the NPE compared to CPE. Grey symbols represent genes expressed significantly higher in the NPE.** Transparent entries are molecules inserted by the knowledge database. Gene names are abbreviated according to those used in GenBank. Solid lines indicate direct physical or functional relationships between molecules (such as regulating and interacting protein domains). The main functionalities given by Ingenuity for this entire molecular network are shown in the diagrams. Reproduced with permission. Click here for file

Additional file 7 **Genes coding ion transport proteins involved in CSF and AH production.** We constructed a list of 157 coding genes corresponding to all the CPE and CBE ion transport proteins as described by Brown *et al*. [30] and Civan *et al.*[20]. Click here for file

Additional file 8 Genes expressed significantly higher in the CPE than in NPE involved in the biological function of angiogenesis. Click here for file
