# Supplementary material for: Gene expression-based comparison of the human secretory neuroepithelia of the brain choroid plexus and the ocular ciliary body: potential implications for glaucoma
Source: Fluids Barriers CNS. 2014 Jan 29;11:2. doi: 10.1186/2045-8118-11-2 (PMC3909915; doi:10.1186/2045-8118-11-2)
Supplement: Additional file 3 — Molecular networks 2–25 generated by the Ingenuity software from the genes expressed significantly higher in the CPE compared to NPE. Grey symbols represent genes expressed significantly higher in the CPE. Transparent entries are molecules inserted by the knowledge database. Gene names are abbreviated according to those used in GenBank. Solid lines indicate direct physical or functional relationships between molecules (such as regulating and interacting protein domains). The main functionalities given by Ingenuity for this entire molecular network are shown in the diagrams. Reproduced with permission. [file 2045-8118-11-2-S3.pdf]

Network 2 - CPE

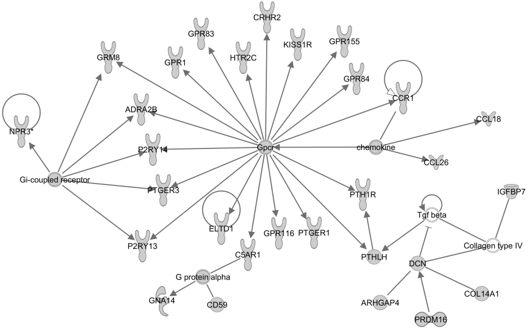


Top functions: Cell to cell signaling and interaction, molecular transport

© 2000-2013 Ingenuity Systems, Inc. All rights reserved.

Network 3 - CPE

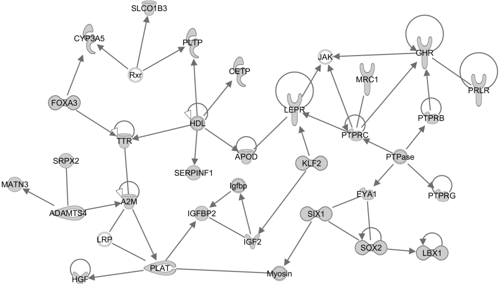


Top functions: Lipid metabolism, molecule transport, small molecule biochemistry

© 2000-2013 Ingenuity Systems, Inc. All rights reserved.

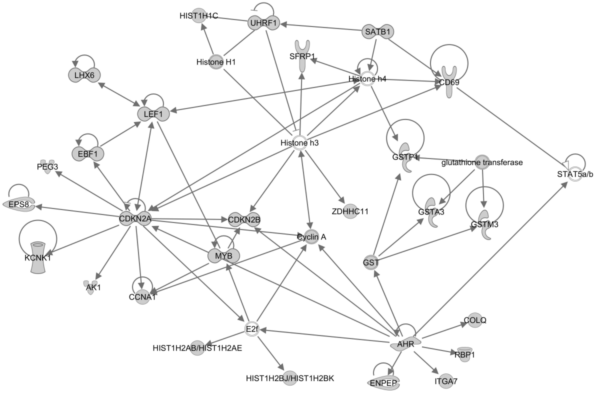


Top functions: Cellular development, hematological system development and function, hematopoiesis

© 2000-2013 Ingenuity Systems, Inc. All rights reserved.

Network 5 - CPE

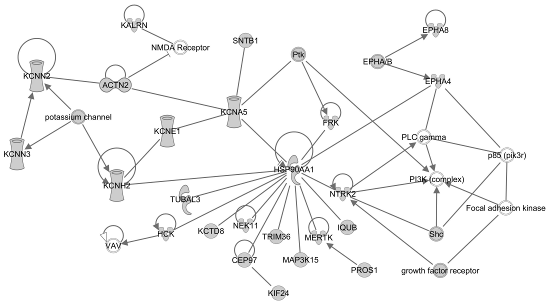


Top functions: Molecular transport, cell to cell signaling and interaction and nervous system development and function

© 2000-2013 Ingenuity Systems, Inc. All rights reserved.

Network 6 - CPE

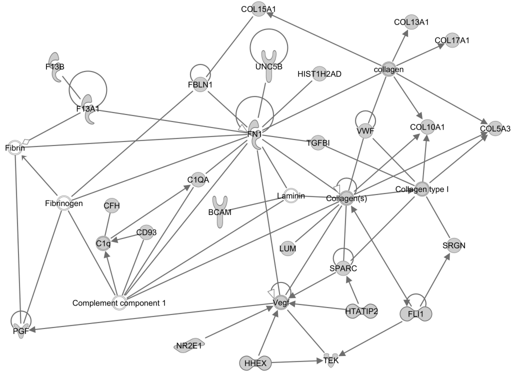


Top functions: Cell to cell signaling and interaction, cellular assembly and organization, tissue development

© 2000-2013 Ingenuity Systems, Inc. All rights reserved.

Network 7 - CPE

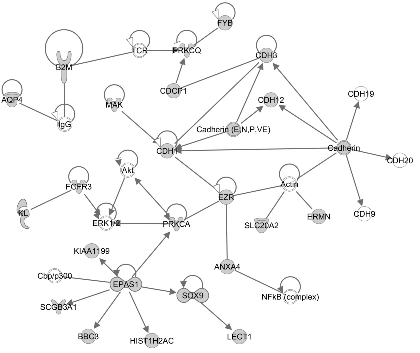


Top functions: Organismal injury and abnormalities, cellular function and maintenance and molecular transport

© 2000-2013 Ingenuity Systems, Inc. All rights reserved.

Network 8 - CPE

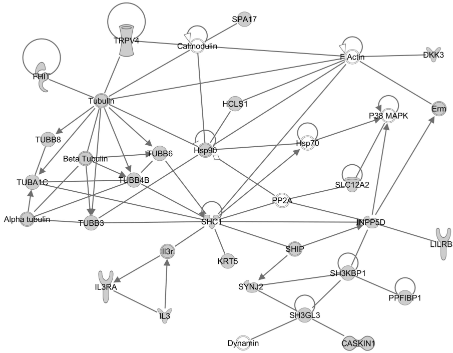


Top functions: Cancer, dermatological disease and conditions, hematological disease

© 2000-2013 Ingenuity Systems, Inc. All rights reserved.

Network 9 - CPE

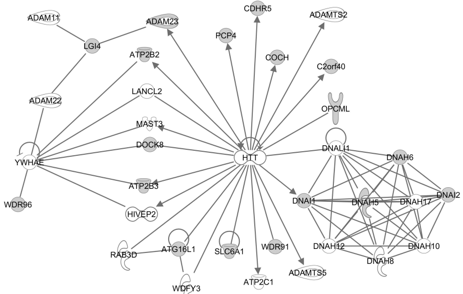


Top functions: Hereditary disorder, respiratory disease, cell signaling

© 2000-2013 Ingenuity Systems, Inc. All rights reserved.

Network 10 - CPE

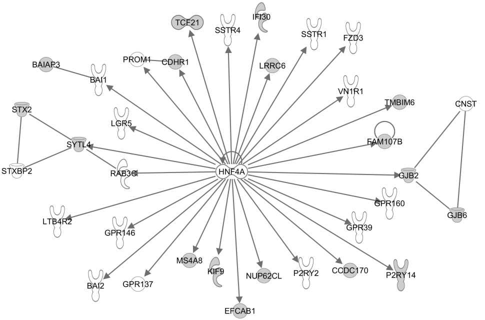


Top functions: Hereditary, auditory and neurological diseases

© 2000-2013 Ingenuity Systems, Inc. All rights reserved.

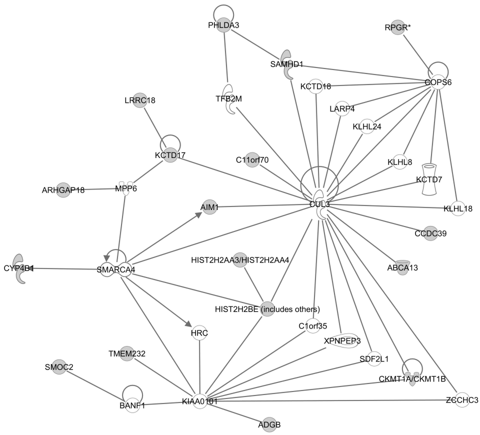


Top functions: Connective tissue disorders, dental disease, developmental disorder

© 2000-2013 Ingenuity Systems, Inc. All rights reserved.

Network 12 - CPE

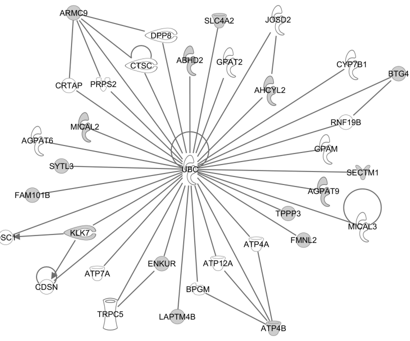


Top functions: Gastointestinal disease, digestive system development and function, organ morphology

© 2000-2013 Ingenuity Systems, Inc. All rights reserved.

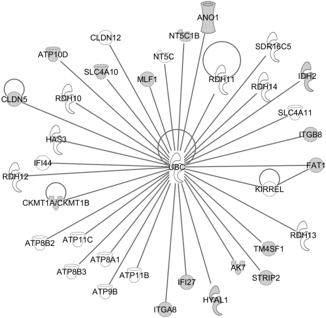


Top functions: Visual system development and function, lipid metabolism, small molecule biochemistry

© 2000-2013 Ingenuity Systems, Inc. All rights reserved.

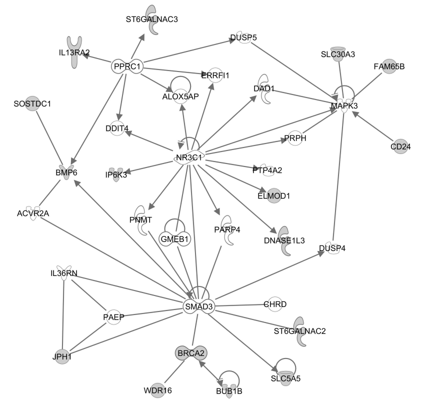


Top functions: Connective tissue disorders, developmental disorder, skeletal and muscular disorders

© 2000-2013 Ingenuity Systems, Inc. All rights reserved.

Network 15 -CPE

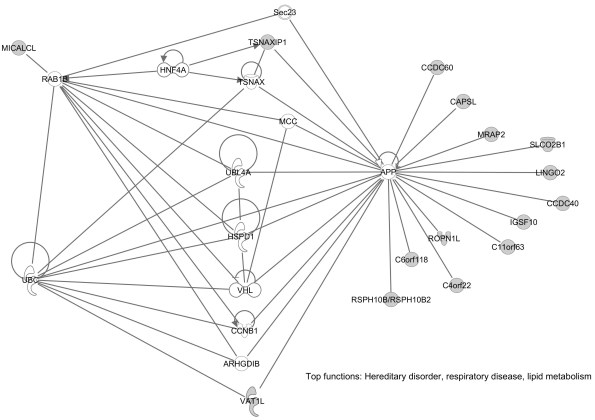


Top functions: Hereditary disorder, respiratory disease, lipid metabolism

© 2000-2013 Ingenuity Systems, Inc. All rights reserved.

Network 16 - CPE

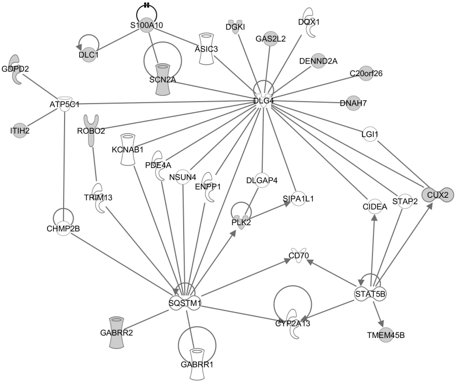


Top functions: Nervous system development and function, nutritional disease, neurological disease

© 2000-2013 Ingenuity Systems, Inc. All rights reserved.

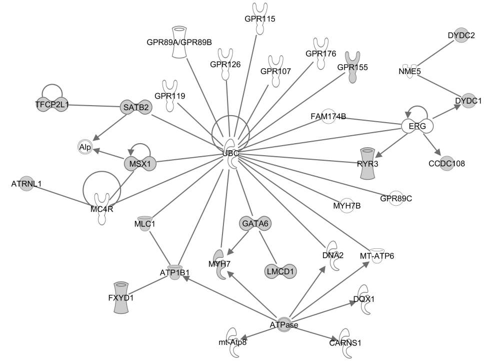


Top functions: DNA replication, recombination, and repair; energy production; nucleic acid metabolism

© 2000-2013 Ingenuity Systems, Inc. All rights reserved.

Network 18 - CPE

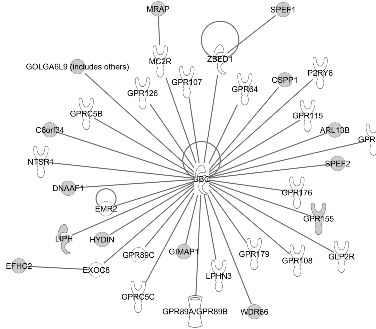


Top functions: Endocrine system disorders, hereditary disorder, respiratory disease

© 2000-2013 Ingenuity Systems, Inc. All rights reserved.

# Network 19 - CPE

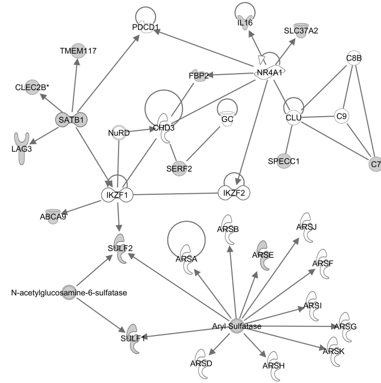


Top functions: Humoral immune response, inflammatory response, carbohydrate metabolism

© 2000-2013 Ingenuity Systems, Inc. All rights reserved.

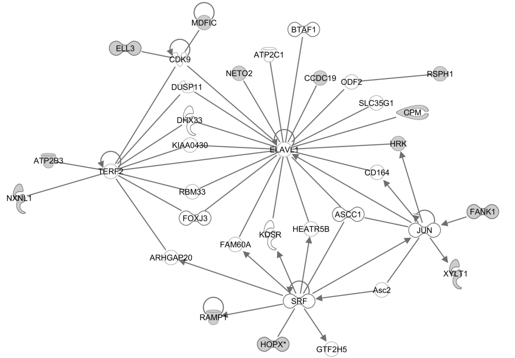


Top functions: Embryonic development, tissue morphology, developmental disorder

© 2000-2013 Ingenuity Systems, Inc. All rights reserved.

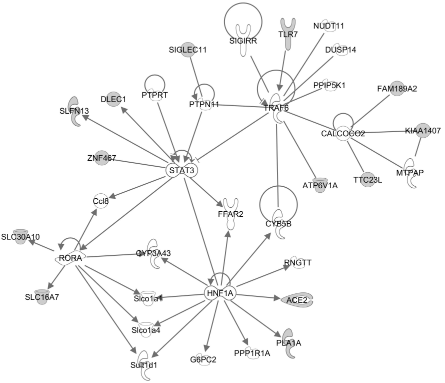


Top functions: Cell mediated immune response, cellular development, cellular function and maintenance

© 2000-2013 Ingenuity Systems, Inc. All rights reserved.

Network 22 - CPE

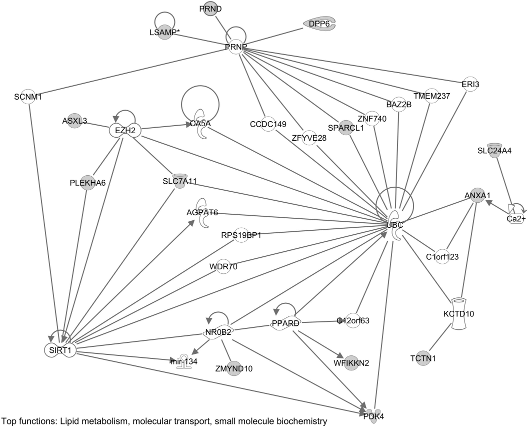


Top functions: Lipid metabolism, molecular transport, small molecule biochemistry

© 2000-2013 Ingenuity Systems, Inc. All rights reserved.

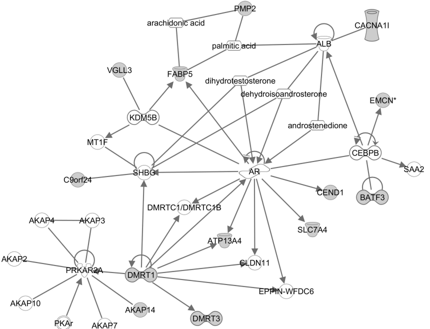


Top functions: Embryonic, organ and organismal development

© 2000-2013 Ingenuity Systems, Inc. All rights reserved.

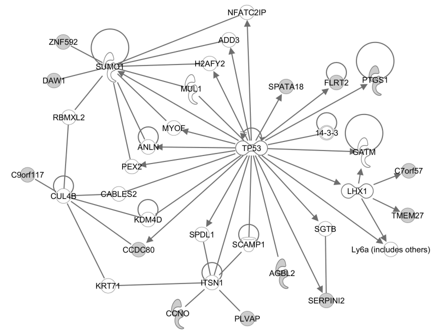


Top functions: Cell cycle, decreased levels of albumin, cell death and survival

© 2000-2013 Ingenuity Systems, Inc. All rights reserved.

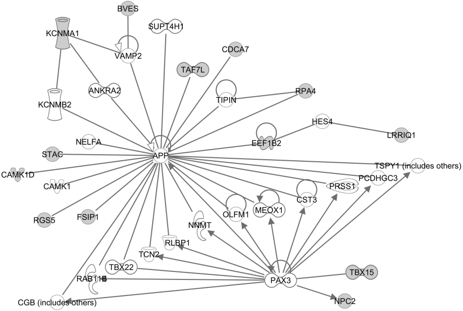


Top functions: Neurological disease, cardiovascular disease, developmental disorder

© 2000-2013 Ingenuity Systems, Inc. All rights reserved.
