# Supplementary material for: Gene expression-based comparison of the human secretory neuroepithelia of the brain choroid plexus and the ocular ciliary body: potential implications for glaucoma
Source: Fluids Barriers CNS. 2014 Jan 29;11:2. doi: 10.1186/2045-8118-11-2 (PMC3909915; doi:10.1186/2045-8118-11-2)
Supplement: Additional file 5 — Genes in the NPE expressed significantly higher than in CPE involved in the biological function of cellular protrusions. [file 2045-8118-11-2-S5.docx]

Additional file 5: Significantly higher expressed genes in the NPE than in CPE involved in the biological function of cellular protrusions

| **Gene name** | **Systemic name** | **Function** |
| --- | --- | --- |
| ADCYAP1 | NM_001117 | Neuroendocrine stress response |
| ANK3 | NM_020987 | Actin cytoskeleton |
| ARHGAP24 | NM_001025616 | Actin remodeling, cell polarity and cell migration |
| ARHGEF28 | AK025816 | Formation of ALS neurofilament aggregates |
| BMP2 | NM_001200 | Bone and cartilage formation |
| CHRNA3 | NM_000743 | Neurotransmission |
| CIT | NM_007174 | Neuronal development |
| CLRN1 | NM_174878 | Homeostasis of retina |
| CNTN4 | NM_175607 | Axon-associated cell adhesion |
| DRD1 | NM_000794 | Dopamine receptor |
| EFNA2 | BX375060 | Neuronal development |
| EFNA5 | BU607658 | Anchor plasmam membrane |
| EGF | NM_001963 | Epithelial growth factor |
| ENC1 | NM_003633 | Actin binding protein |
| FGF13 | AI762428 | Fibroblast growth factor |
| FGFR1 | NM_023110 | Fibroblast growth factor receptor |
| GAP43 | NM_002045 | Axonal development and regeneration |
| GJA1 | NM_000165 | Gap junction |
| GRIN3A | NM_133445 | Glutamate-gated ion channel |
| GRP | NM_002091 | Neuro-endocrine peptide |
| INHBA | NM_002192 | Neuro-endocrine peptide |
| LRRC4C | NM_020929 | Axom guidance |
| MARCKS | NM_002356 | Actin filament crosslinking protein |
| MCF2 | NM_005369 | Regulation of Rho |
| NTF3 | NM_002527 | Survival and differentiation of neurons |
| PALLD | NM_016081 | Actin cytoskeleton |
| PAX6 | NM_000280 | Development |
| PLCG2 | NM_002661 | Signaling enzyme |
| PLD1 | NM_002662 | Catalytic activity |
| PVRL3 | BC017572 | Adhesion molecule at adherens junctions |
| RELN | NM_005045 | Extracellular matrix protein involved in cell-cell interactions and neuronal migration |
| RHOU | NM_021205 | Rho family involved in filopodium formation and stress fiber dissolution; cytoskeleton organization |
| S100A4 | NM_002961 | Motility, invasion and tubulin polymerization |
| SCN4B | NM_174934 | Ion-channel |
| SEMA3A | NM_006080 | Axonal growth and apical dendrites |
| SEMA3D | NM_152754 | Axonal growth and apical dendrites |
| SKIL | H40632 | Cell growth and differentiation |
| SLIT1 | ENST00000266058 | Axonal guidance |
| SLITRK5 | NM_015567 | Membrane protein |
| ST8SIA1 | NM_003034 | Cell adhesion |
| STXBP5 | BU507302 | Docking and fusion of synaptic vesicles |
| TENM4 | AB037723 |  |
| TGFB3 | NM_003239 | Embryohenesis and cell diffentiation |
| TIAM1 | BX414807 | Axogenesis |
| TIAM2 | NM_012454 | Neural cell development |
| TPBG | ENST00000369750 | Cell adhesion |
| UGCG | AI824599 | Membrane components |
