# Supplementary material for: Gene expression-based comparison of the human secretory neuroepithelia of the brain choroid plexus and the ocular ciliary body: potential implications for glaucoma
Source: Fluids Barriers CNS. 2014 Jan 29;11:2. doi: 10.1186/2045-8118-11-2 (PMC3909915; doi:10.1186/2045-8118-11-2)
Supplement: Additional file 6 — Molecular networks generated by the Ingenuity software from the genes expressed significantly higher in the NPE compared to CPE. Grey symbols represent genes expressed significantly higher in the NPE. Transparent entries are molecules inserted by the knowledge database. Gene names are abbreviated according to those used in GenBank. Solid lines indicate direct physical or functional relationships between molecules (such as regulating and interacting protein domains). The main functionalities given by Ingenuity for this entire molecular network are shown in the diagrams. Reproduced with permission. [file 2045-8118-11-2-S6.pdf]

Network 2 - NPE

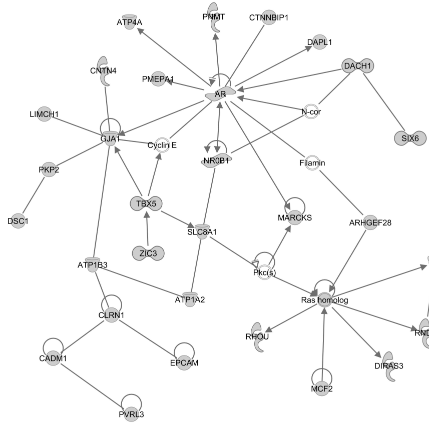

Top functions: Cellular function and maintenance, cellular movement and reproductive system development and function

© 2000-2013 Ingenuity Systems, Inc. All rights reserved.

Network 3 - NPE

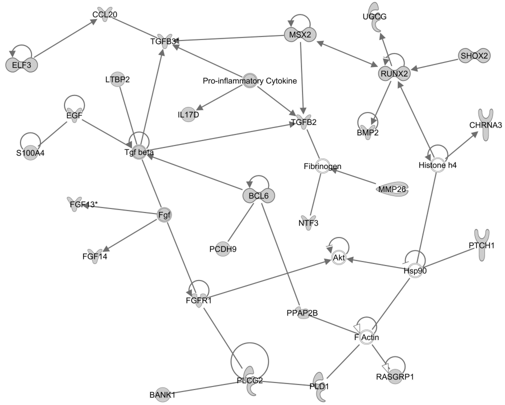

Top functions: Skeletal and muscular system development and function, tissue and cellular development

© 2000-2013 Ingenuity Systems, Inc. All rights reserved.

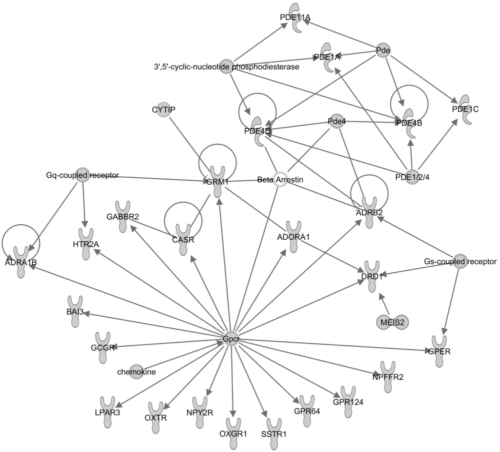

Top functions: Cell to cell signaling and interaction, psychological disorders, cell signaling

© 2000-2013 Ingenuity Systems, Inc. All rights reserved.

Network 5 - NPE

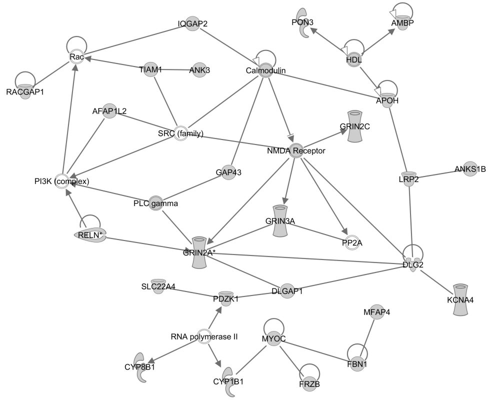

Top functions: Ophthalmic, respiratory and neurological disease

© 2000-2013 Ingenuity Systems, Inc. All rights reserved.

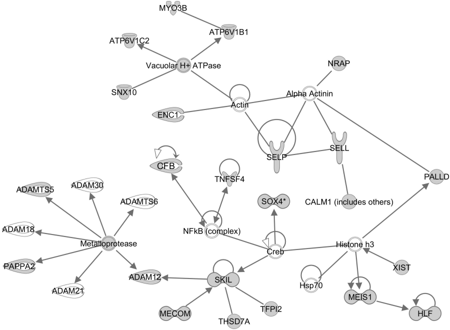

Top functions: Cellular movement, hematological system development and function, immune cell trafficking

© 2000-2013 Ingenuity Systems, Inc. All rights reserved.

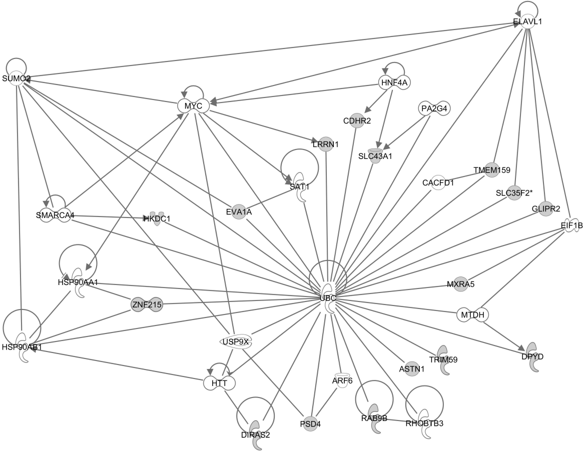

Top functions: Molecule transport, small molecule biochemistry and cell death and survival

© 2000-2013 Ingenuity Systems, Inc. All rights reserved.

Network 8 - NPE

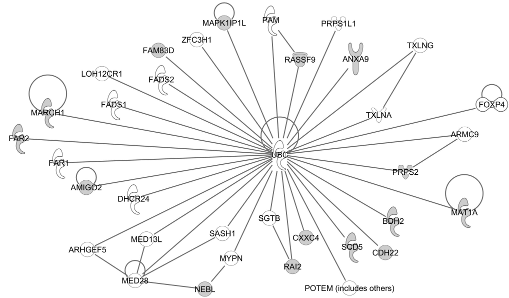

Top functions: Lipid metabolism, small molecule biochemistry and dermatological disease and conditions

© 2000-2013 Ingenuity Systems, Inc. All rights reserved.

Network 9 - NPE

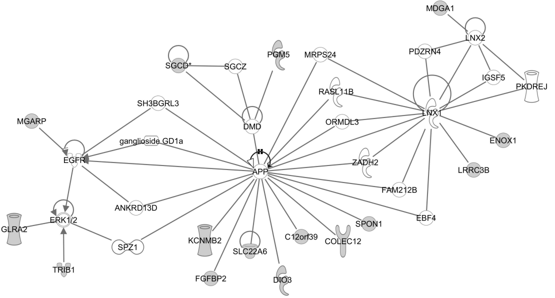

Top functions: Developmental disorder, cell to cell signaling and interaction, nervous system development and function

© 2000-2013 Ingenuity Systems, Inc. All rights reserved.

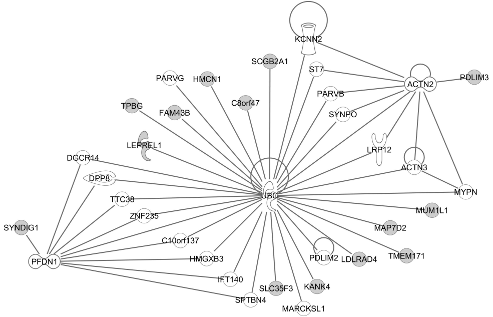

Top functions: Hereditary disorder, ophthalmic disease, cardiovascular disease

© 2000-2013 Ingenuity Systems, Inc. All rights reserved.

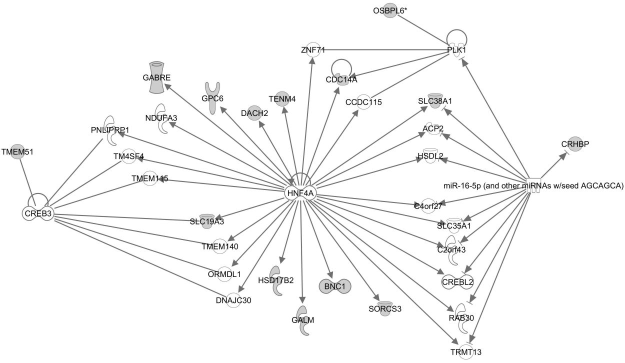

Top functions: Cellular assembly and organization, DNA replication, recombination, and repair and neurological disease

© 2000-2013 Ingenuity Systems, Inc. All rights reserved.

Network 12 - NPE

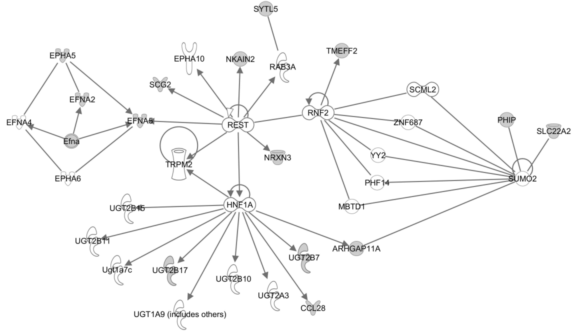

Top functions: Lipid metabolism, small molecule biochemistry, endocrine system development and function

© 2000-2013 Ingenuity Systems, Inc. All rights reserved.

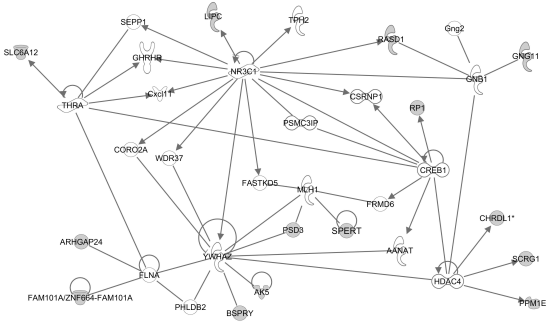

Top functions: Amino acid metabolism, small molecule biochemistry and behavior

© 2000-2013 Ingenuity Systems, Inc. All rights reserved.

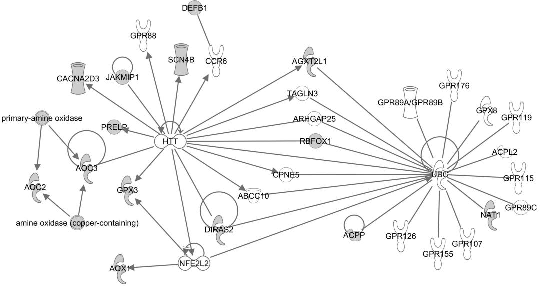

Top functions: Hereditary disorder, neurological disease and psychological disorders

© 2000-2013 Ingenuity Systems, Inc. All rights reserved.

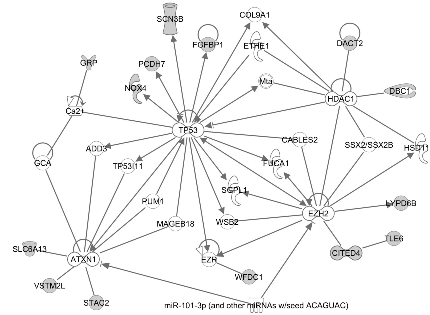

Top functions: Developmental disorder, endocrine system disorders and reproductive system disease

© 2000-2013 Ingenuity Systems, Inc. All rights reserved.

Network 16 - NPE

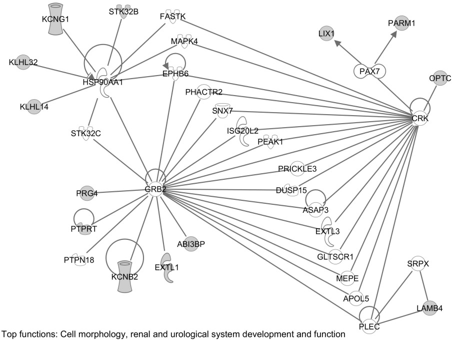

Top functions: Cell morphology, renal and urological system development and function

© 2000-2013 Ingenuity Systems, Inc. All rights reserved.

Network 17 - NPE

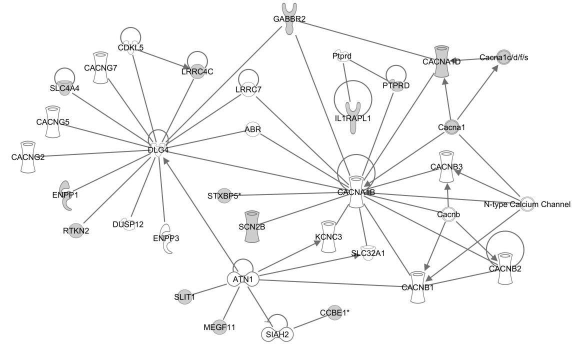

Top functions: Cardiovascular disease, organismal injury and abnormalities and cardiac arteriopathy

© 2000-2013 Ingenuity Systems, Inc. All rights reserved.

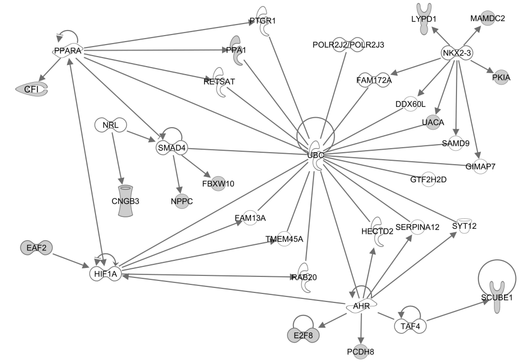

Top functions: Cancer, gastrointestinal and hepatic system disease

© 2000-2013 Ingenuity Systems, Inc. All rights reserved.

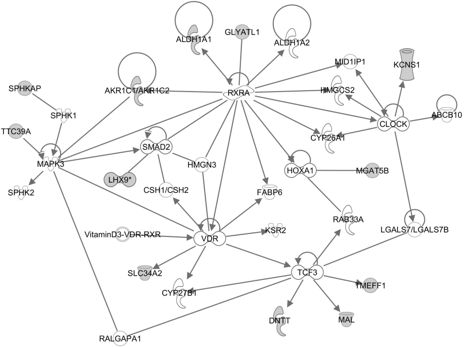

Top functions: Vitamin, mineral, drug and lipid metabolism

© 2000-2013 Ingenuity Systems, Inc. All rights reserved.

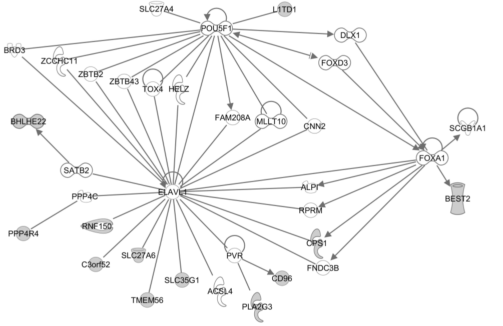

Top functions: Dermatological diseases, lipid and small molecule metabolism

© 2000-2013 Ingenuity Systems, Inc. All rights reserved.

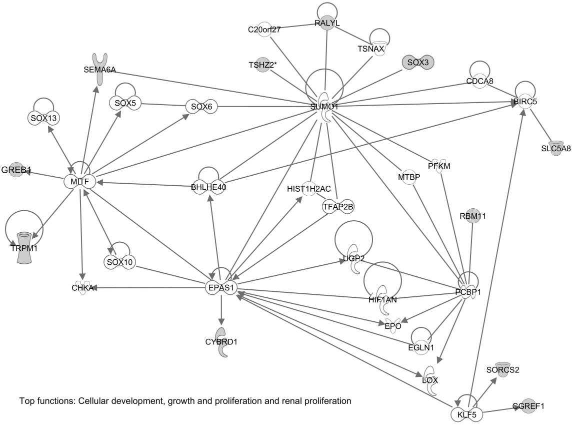

Top functions: Cellular development, growth and proliferation and renal proliferation

© 2000-2013 Ingenuity Systems, Inc. All rights reserved.

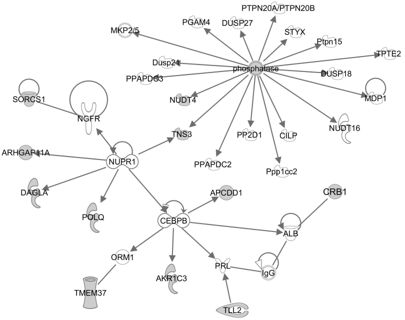

Top functions: Cell to cell signaling and interaction, lipid metabolism, reproductive system development and function

© 2000-2013 Ingenuity Systems, Inc. All rights reserved.

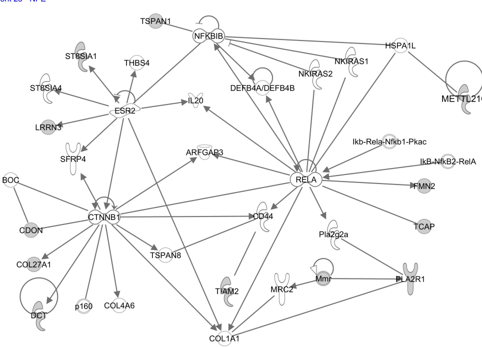

Top functions: Connective tissue, skeletal, muscular and dermatological disorders

© 2000-2013 Ingenuity Systems, Inc. All rights reserved.

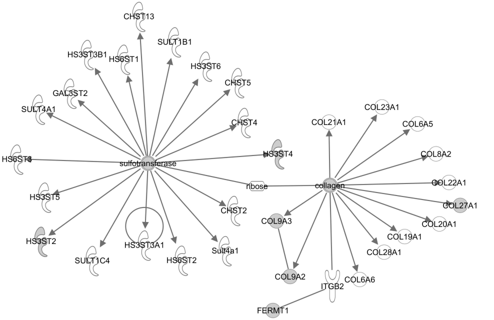

Top functions: Connective tissue disorders, dermatological and gastointestinal diseases

© 2000-2013 Ingenuity Systems, Inc. All rights reserved.

Network 25 - NPE

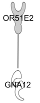

Top functions: Cancer and cellular movement

© 2000-2013 Ingenuity Systems, Inc. All rights reserved.
