# Supplementary material for: Gene expression-based comparison of the human secretory neuroepithelia of the brain choroid plexus and the ocular ciliary body: potential implications for glaucoma
Source: Fluids Barriers CNS. 2014 Jan 29;11:2. doi: 10.1186/2045-8118-11-2 (PMC3909915; doi:10.1186/2045-8118-11-2)
Supplement: Additional file 8 — Genes expressed significantly higher in the CPE than in NPE involved in the biological function of angiogenesis. [file 2045-8118-11-2-S8.docx]

Supplementary Table 4: Specifically expressed CPE genes compared NPE involved in the biological function of angiogenesis

| **Gene name** | **Systemic name** | **Gene name** | **Systemic name** |
| --- | --- | --- | --- |
| ACE2 | NM_021804 | KLF2 | NM_016270 |
| ADRA2B | NM_000682 | LECT1 | NM_007015 |
| AHR | NM_001621 | LEF1 | NM_016269 |
| BMP6 | NM_001718 | LMO2 | NM_005574 |
| CCL26 | NM_006072 | NR2E1 | NM_003269 |
| CDKN2A | NM_058197 | NTRK2 | NM_001007097 |
| COL13A1 | NM_005203 | PGF | NM_002632 |
| COL15A1 | NM_001855 | PLAT | NM_000930 |
| CRHR2 | Y10152 | PLVAP | NM_031310 |
| DCN | NM_001920 | PRKCA | NM_002737 |
| EPAS1 | NM_001430 | PTGER3 | NM_198715 |
| EYA1 | NM_000503 | PTH1R | NM_000316 |
| FABP4 | W60781 | PTHLH | ENST00000354417 |
| FBLN1 | NM_006486 | PTPRB | AK097371 |
| FGFR3 | NM_000142 | RGS5 | NM_003617 |
| FN1 | NM_212482 | SELE | NM_000450 |
| FOXC1 | NM_001453 | SERPIND1 | NM_000185 |
| GATA6 | NM_005257 | SERPINF1 | NM_002615 |
| HCK | NM_002110 | SHC1 | NM_183001 |
| HGF | ENST00000222390 | SMOC2 | NM_022138 |
| HHEX | NM_002729 | SOX17 | NM_022454 |
| HTATIP2 | AF092095 | SPARC | NM_003118 |
| IGF2 | NM_000612 | SRPX2 | NM_014467 |
| IGFBP7 | NM_001553 | SULF1 | NM_015170 |
| IL13RA2 | NM_000640 | TEK | NM_000459 |
| IL3 | NM_000588 | TGFBI | NM_000358 |
| ITGA7 | NM_002206 | TM4SF1 | NM_014220 |
| ITGB8 | NM_002214 | UNC5B | NM_170744 |
| KCNMA1 | NM_002247 |  |  |
